# Shear-Wave Elastography and Viscosity PLUS for the Assessment of Peripheric Muscles in Healthy Subjects: A Pre- and Post-Contraction Study

**DOI:** 10.3390/diagnostics12092138

**Published:** 2022-09-02

**Authors:** Ioana-Teofana Dulgheriu, Carolina Solomon, Delia Doris Muntean, Raluca Petea-Balea, Manuela Lenghel, Anca Ileana Ciurea, Sorin Marian Dudea

**Affiliations:** Department of Radiology, “Iuliu Hatieganu” University of Medicine and Pharmacy, 400012 Cluj-Napoca, Romania

**Keywords:** musculoskeletal ultrasound, elastography, viscoelasticity, ViPLUS, novel techniques

## Abstract

Viscosity is a novel parameter, recently introduced in the use of elastographic techniques, correlating to shear-wave dispersion. The purpose of this study was to provide normal reference viscosity values for the peripheral muscles in healthy volunteers. This prospective study included 38 subjects who underwent US examinations between November 2021 and January 2022. Measurements were taken on the calf and the deltoid muscles in both pre- and post-contraction states. The age range was 21–29 years, with a median of 26 years. The SWE and ViPLUS values in the deltoid muscles were significantly higher than in the soleus muscles in both pre- and post-contraction sets (*p* = 0.002). There were statistically significant differences between the pre- and post-contraction values for both the SWE and ViPLUS values in the subgroup analysis. The ICC estimates and the 95% confidence intervals were based on a mean rating (k = 2), an absolute agreement, and a two-way random-effects model, demonstrating excellent agreement between the measurements taken by the two examiners.

## 1. Introduction

Elastography is a novel, non-invasive ultrasound application used to assess tissue stiffness. The values obtained can be qualitative or quantitative, differing in the underlying physical mechanism and further dividing the method into the following two categories: strain elastography and shear-wave elastography (SWE). Shear-wave velocities are measured after the propagation of high-frequency acoustic pulses in a transverse direction from the transducer [1]. The general principle is that shear waves move faster through more rigid tissues. The tissue rigidity is expressed in absolute values, meters per second (m/s), or calculated using the Young’s modulus in kilopascals (kPa). In order to calculate Young’s modulus, the machine assumes that the tissue density is linear, constant, and isotropic [2]. However, soft tissues are nonlinear, heterogeneous, anisotropic, and viscoelastic. Viscosity is an ignored parameter in the current use of elastographic techniques. Viscosity correlates to the shear-wave dispersion [3]. Various methods have been developed to assess the tissue viscoelastic properties by measuring the shear-wave dispersion and attenuation, especially in dedicated phantom models or in the liver [4,5,6,7,8,9].

Anisotropy is a well-known property of skeletal muscles that depends on the angle of insonation of the incident ultrasound beam. Additionally, the skeletal muscle tissue is a viscous material, and this feature has an effect on the mechanics of passive muscle extensions. The behavior of viscoelastic materials under uniaxial loading has been long represented by different conceptual models, known as rheological or mechanical models, such as the Maxwell model or the Voigt model [10]. Adding viscosity to the medium will also affect the tissue stiffness measured as an effect of dispersion. Isotropy and homogeneity assumptions can also be broken at tissue interfaces, where wave reflection can cause erroneous measurements. As Van Loocke et al. [11] and Wheatley et al. [12] showed in their proposed constitutive models, the viscoelastic component plays an important role in muscle mechanics. The elastic behavior of muscles is nonlinear and transversely isotropic, with the stiffest direction in the fiber direction [11]. Chen et al. [13] presented a model to measure viscosity using ultrasound radiation force to generate cylindrical shear waves of certain frequencies in dedicated phantoms. As Romano et al. [14] correctly observed, there are numerous protocols and general factors that prevent the standardization of the muscle SWE measurements.

Many manufacturers have developed elastography techniques for their machines, but no consensus concerning the measurements and cut-offs exists. A great range of normal SWE values are reported but without clear reference values.

Studies on muscle elastography suggest that this technique can provide important information on the mechanical properties of muscles and detect particular changes in different pathologies [15,16,17]. Elastography can quantify alterations related to inflammation, degeneration, injury, healing, and treatment response [1,18]. 

The EFSUMB Guidelines and Recommendations mention an increase in the number of studies using musculoskeletal elastography and recommend it as an alternative to electromyography in neurologic disorders or as a complementary method in the diagnosis and surveillance of inflammatory myopathies [19]. There are also studies that show statistically significant differences in the values of contracted muscles compared to their relaxed state [16]. A few studies exist on the viscoelastic properties of muscles [20,21,22].

The purpose of this study was to provide normal reference viscosity values for the peripheral muscles in healthy volunteers. The primary objective was to assess the existence of different viscosity values in muscles in pre- and post-contraction states. The secondary objective was to compare the SWE and the Viscosity Plane-Wave Ultrasound (ViPLUS) values in order to evaluate the correlation between the two methods and to analyze the inter-observer variability of the values on the same machine. To the best of our knowledge, the present study is the first to attempt to provide normal muscle reference viscosity values.

## 2. Materials and Methods

A prospective, monocentric study was performed between November 2021 and January 2022. The study comprised 38 healthy and young volunteers, with no known muscular pathologies. Subjects with neuromuscular diseases or musculoskeletal injuries were excluded. The images were obtained on an Aixplorer MACH^®^ ultrasound machine (Supersonic Imagine, Aix-en-Provence, France) with a curvilinear transducer model C6-1X using a B-mode ultrasound, SWE elastography, and ViPLUS. The imaging parameters were as follows: maximum transducer frequency available; optimization, resolution; persistence, medium; color box, minimum dimensions available; ROI diameter, 5 mm; depth, 2 cm. The transducer used was the only one with a ViPLUS mode available.

### 2.1. Image Acquisition

**Figure 1:**
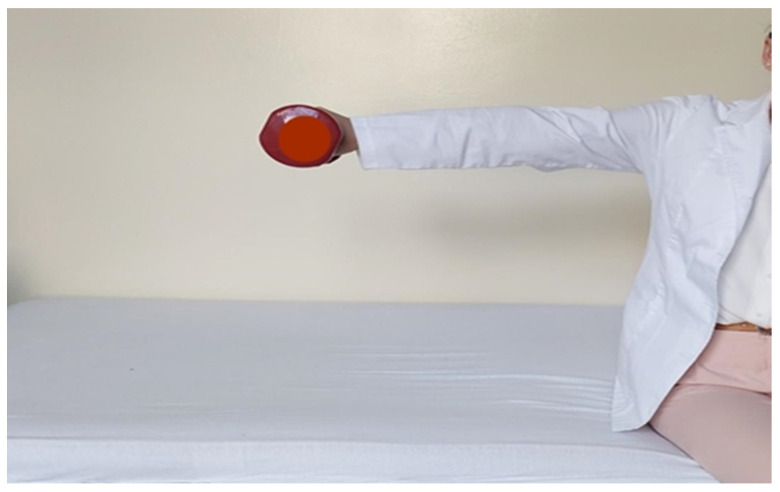
Sustained (30-s) deltoid contraction obtained by holding a 3-kg dumbbell weight.

**Figure 2:**
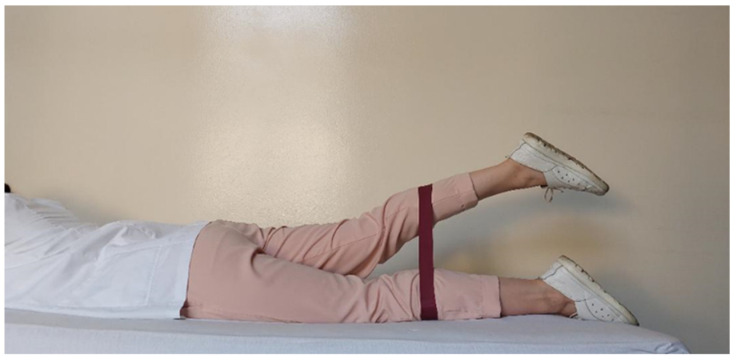
Sustained (30-s) calf muscle contraction obtained by pointing the toes forward, resisting the elastic band, and holding the tension.

Two examiners with five and four years of experience in ultrasound, respectively, performed the measurements for each subject on the same day with a 15-min pause between the measurement sets. The subjects were instructed to refrain from performing physical activity for 24 h prior to the examination. Before the examination, the subjects were asked to rest for 30 min and adjust to the room temperature. Measurements were made on the right calf (soleus) and the right deltoid muscles of each volunteer. For the deltoid muscle, a relaxing position was defined as a flexed elbow and the arm resting on a pillow. For the calf measurements, the subjects were laid in a prone position with their knees fully extended. Firstly, the transducer was oriented parallel to the longitudinal axis of the muscle fibers and an optimal B-mode image was obtained. Then, the SWE and ViPLUS modes were activated with minimum compression used, avoiding vessels and interfascial planes. The locations that achieved less than a 90% stability index (SI) or more than a 10% standard deviation were rejected. The acquisition depths were set at 2 to 2.5 cm from the skin surface for the deltoid and at 3–4 cm from the skin in the midportion of the muscle belly for the soleus muscles. The elastographic color box was adjusted to the minimum dimensions available and placed in the center of the image with the regions of interest (ROIs) in the center. Following the stabilization of the hand, the probe, and the image for 3–5 s, the values were measured as follows: three measurements in a neutral position with relaxed muscles and three measurements after 30 s of sustained continuous contractions. To obtain a homogenous, reproducible contraction in all subjects, the contraction was obtained by holding a 3-kg dumbbell weight in a 90-degree horizontal position for the deltoid muscle (as shown in Figure 1) and by pointing the toes forward whilst resisting an elastic band and holding the tension, for the calf muscles (Figure 2). The SWE measurements were expressed in kilopascals (kPa) as a mean and standard deviation (SD). The ViPLUS mode provides a color-coded map and a quantitative expression of the values in pascal-seconds (Pa.s) as a mean, median, minimum, maximum, and SD. Underneath these values, the depth of the Qbox, the ROI diameter, and the SI are shown. The mean values of the consecutive measurements obtained were used to assess the muscle rigidity and viscosity.

### 2.2. Statistical Analysis

The statistical analysis was conducted with the MedCalc Statistical Software version 20 (MedCalc Software Corp., Brunswick, ME, USA) and the GraphPad Prism v. 8.0.1 for Windows (GraphPad Software, San Diego, CA, USA) at a significance level of 5%.

The Kolmogorov–Smirnov and Shapiro–Wilk tests were used to evaluate the normality of the data. The normally distributed quantitative data were presented as a mean ± standard deviation (SD). The non-normally distributed data were presented as a median and range. A paired t-test was used to assess for differences in the means between the subgroups. The Wilcoxon matched-pairs signed-rank test was used for the non-normally distributed data. The relationship between the SWE data and the ViPLUS was investigated using the Pearson’s correlation coefficient, and the Spearman rank correlation was used for the non-normal distribution. The size of the correlation was interpreted as very low (<0.19), low (0.2–0.39), moderate (0.4–0.59), high (0.6–0.79), and very high (0.8–1.0). A one-way analysis of variance (ANOVA) was performed to compare the effects of sex or body mass index (BMI) on the SWE and ViPLUS measurements.

The reproducibility was assessed by determining the ICCs (Intraclass Correlation Coefficients) as follows: a low level of agreement is close to 0, and a high level of agreement tends to be 1. ICC values of ≥0.9 indicate excellent reliability; values between 0.75 and 0.9 indicate good reliability; values between 0.5 and 0.75 indicate moderate reliability; values less than 0.5 are indicative of poor reliability.

## 3. Results

**Figure 3:**
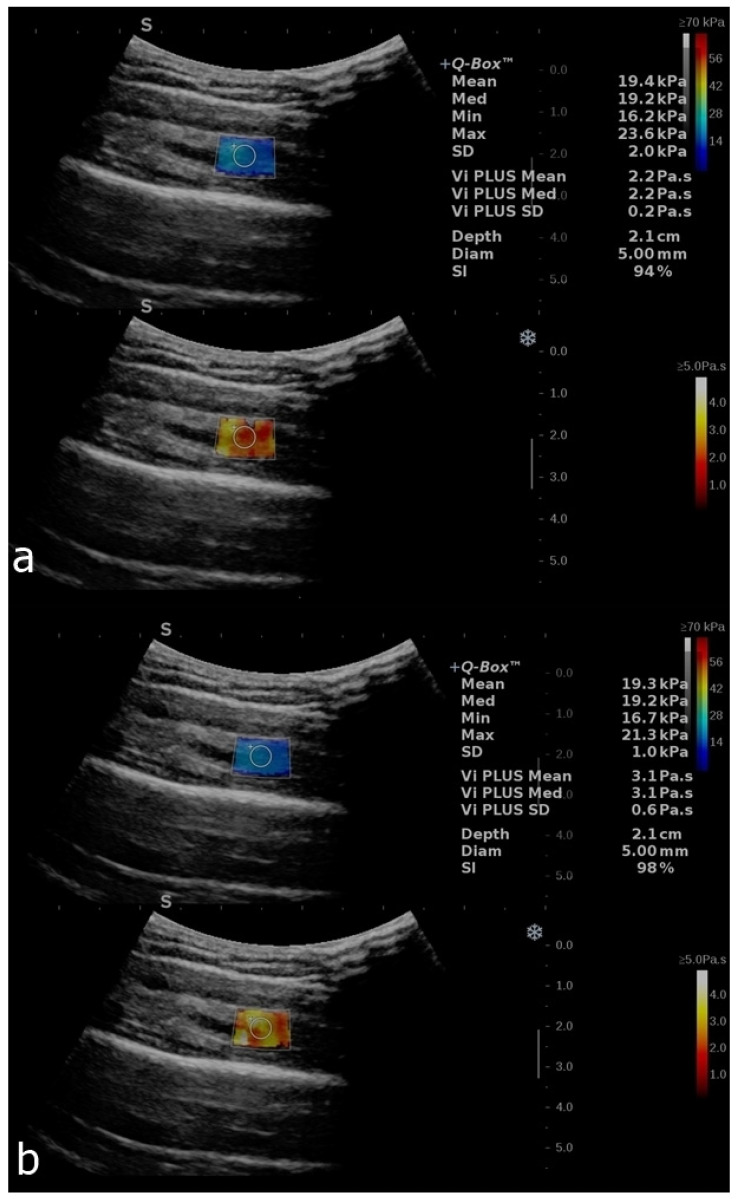
B-mode image with an elastogram of the deltoid muscle in a healthy volunteer. Longitudinal plans of the deltoid muscle as follows: relaxed (**a**) and after contraction (**b**). The regions of interest (ROIs) were placed in the box when the homogenous coloring of the box was obtained with a stability index of over 90%. The top image in each set reflects the SWE mode, and the bottom image reflects the ViPLUS application. ROI values are expressed in kPa for SWE and Pa.s for ViPLUS.

**Figure 4:**
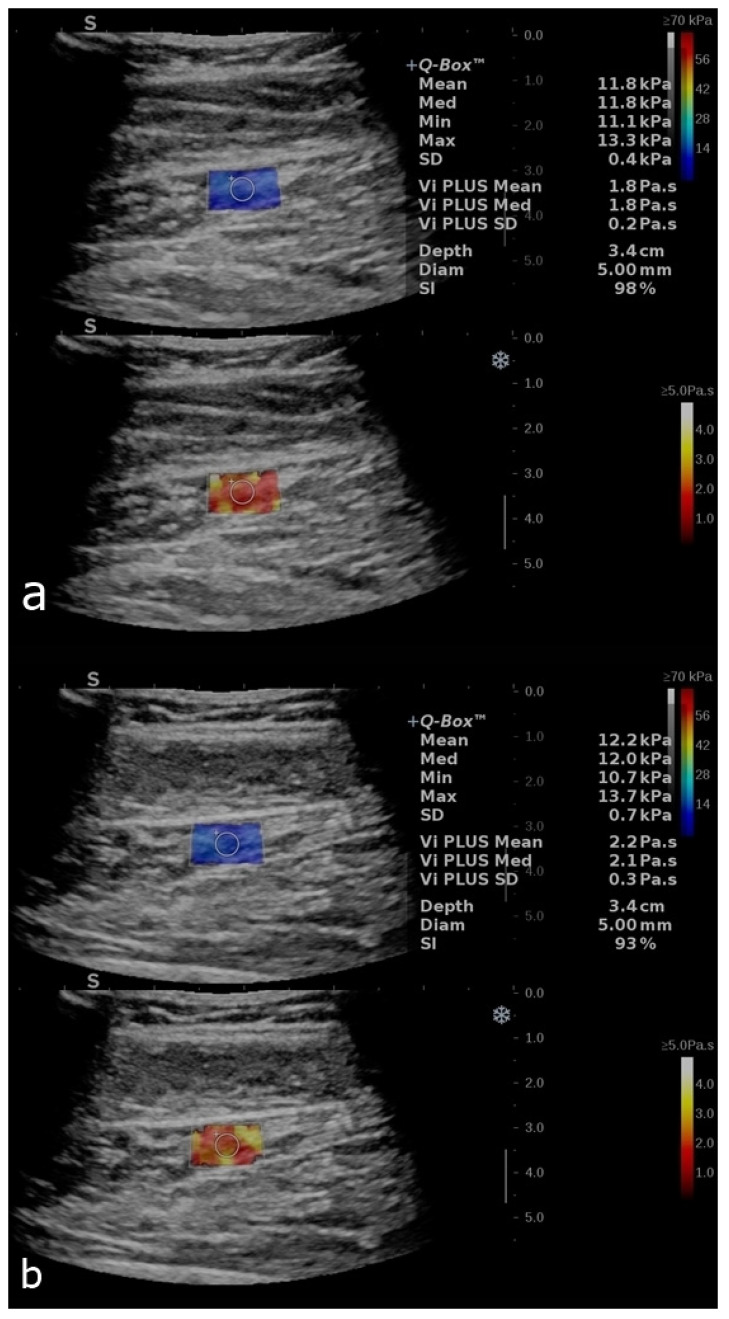
B-mode image with an elastogram of the calf muscle in a healthy volunteer. Longitudinal plans of the calf muscles as follows: relaxed (**a**) and after contraction (**b**). The regions of interest (ROIs) were placed in the box when the homogenous coloring of the box was obtained with a stability index of over 90%. The top image reflects the SWE mode, and the bottom image reflects the ViPLUS application. ROI values are expressed in kPa for SWE and Pa.s for ViPLUS.

Thirty-eight healthy subjects were studied (30 women and 8 men; age range = 21–29 years; median = 26 years). The median BMI was 20.98 (ranging from 17.85 to 34.88). The main demographic characteristics are presented in Table 1. A one-way ANOVA revealed that there were no statistically significant differences in the SWE and ViPLUS measurements related to gender (*p* = 0.518). Figure 3 and Figure 4 show examples of the measurements in different subjects with all of the parameters described accordingly.

**Figure 5:**
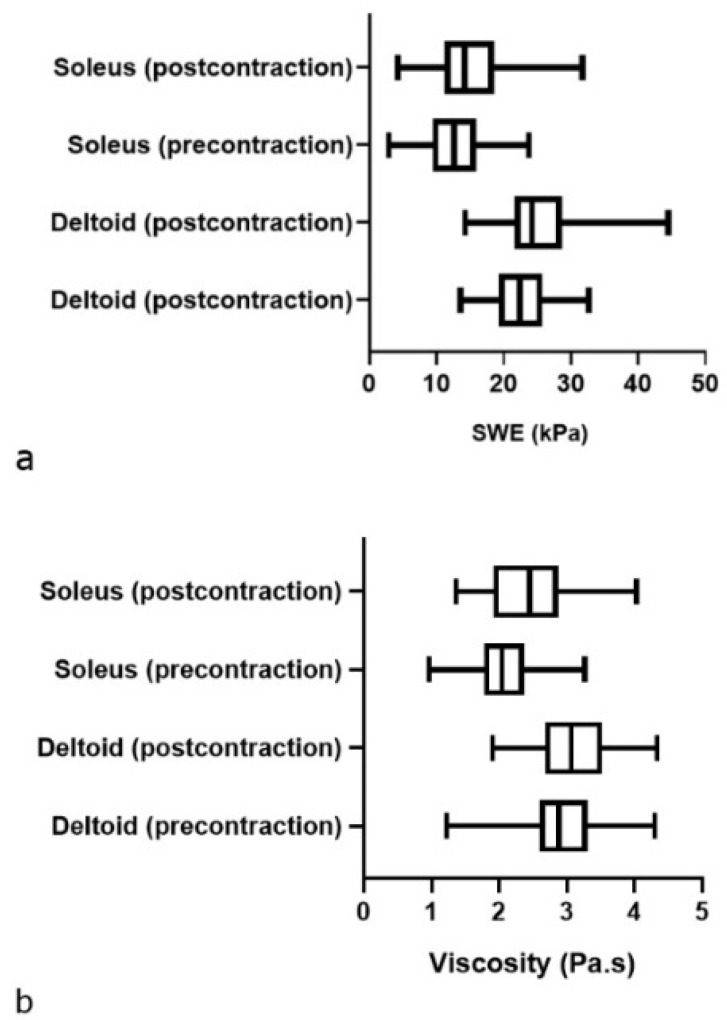
Boxplot showing the tissue elasticity (**a**) and viscosity (**b**) of the deltoid and soleus muscles measured in the longitudinal plane before and after contraction. *p* < 0.05, a significant difference between the two sets of measurements.

Table 2 provides the muscle measurements in both the pre- and post-contraction sets. The SWE and ViPLUS values in the deltoid muscles were significantly higher than in the soleus muscles in both the pre- and post-contraction sets (*p* = 0.002). The same trend was observed for the ViPLUS values, as shown in Figure 5.

There were statistically significant differences between the pre- and post-contraction values in both the SWE and ViPLUS in the subgroup analysis, shown in Table 3.

**Figure 6:**
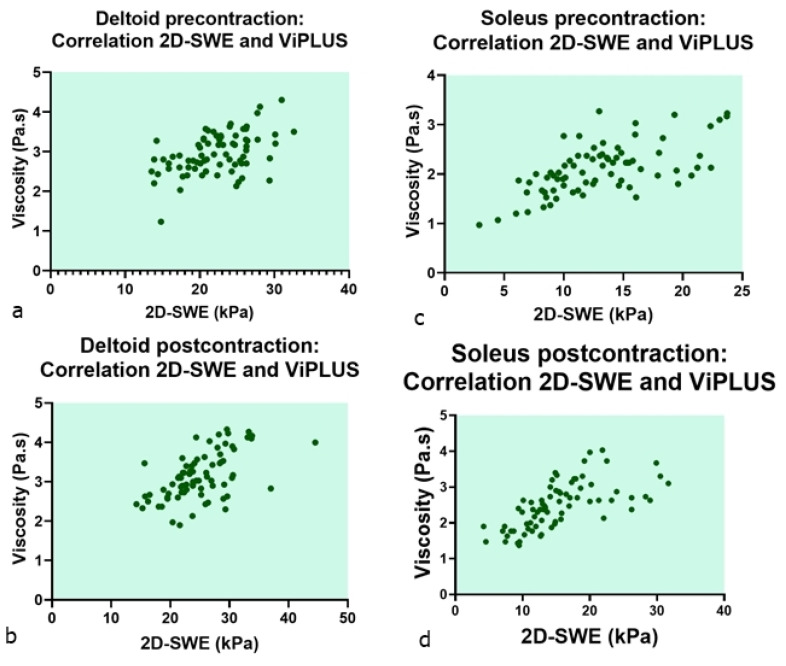
Scatter plot observing the relationship between the elastographic and viscosity data in both muscle groups in pre- (**a**,**c**) and post-contraction (**b**,**d**), showing a moderate to high positive correlation.

There was a moderate to high correlation between the SWE and ViPLUS values in all subgroups, shown in Table 4 and Figure 6.

The ICC estimates and the 95% confidence intervals were based on a mean rating (k = 2), an absolute agreement, and a two-way random-effects model, as shown in Table 5, demonstrating excellent agreement between the measurements taken by the two examiners.

## 4. Discussion

The purpose of the present study was to provide normal reference viscosity values for two different muscle groups in a relaxed and post-contraction status (Table 2). We also measured the normal SWE values, which are close to the values obtained in the literature. For example, for the relaxed soleus muscles, our mean value was 13.1 ± 4.7 kPa. Akkoc et al. [24] obtained a value of 13.4 ± 3.5 kPa and Ferraioli et al. [25] obtained a value of 14.5 kPa.

Our primary objective was met; we found statistically significant differences in the subgroup analysis between the pre- and post-contraction sets. We also found higher values in the deltoid muscle compared to the soleus muscle in both the ViPLUS and the SWE values (Table 3; Figure 5). The boxplot in Figure 5 provides a visual summary of the data, easily identifying the mean values, the dispersion of the sets, and the occasional skewness. In the viscosity boxplot for the soleus group, the median line in the post-contraction group lies outside of the pre-contraction box, showing a likely difference between the two groups. In all of the other boxes, the median line does not lie outside of the comparison box but corresponds to a statistically significant different value in each case. The box lengths (the interquartile ranges) tend to vary more in the viscosity boxplot, suggesting more dispersed data.

Muscle stretching could be an explanation for the increase in muscle stiffness. Our results support other published observations in the literature, which show an increase in stiffness in post-exercise values compared with the pre-exercise values [16]. Nakamura et al. [26] observed that stretching for more than 2 min decreases muscle stiffness. In our study, the second set of measurements was taken immediately after a 30-s contraction. Gennisson et al. [20] assessed the anisotropic nature of the muscles by using SWE during muscle contractions, as well as in a passive extension. Chen et al. [13] presented a model to measure viscosity using an ultrasound radiation force but observed that tissue inhomogeneity can cause a reflection of the shear waves and, consequently, imprecise measurements. Studies [27] performed on the active stretching of the muscles have shown a linear increase between the SWE measurements and the progressive contractions. The same principle was observed in our study, with statistically significant greater values in both the SWE and ViPLUS modes. The reliability appears to be higher for superficial muscles in comparison to deeper muscles as the attenuation of the acoustic pulses and tracking waves increases at greater depths [14].

The ViPLUS modulus provided by the Aixplorer MACH 30 system analyzes the shear-wave propagation speed in order to give information concerning shear-wave dispersion in the tissues. Concerning our secondary objective, the relationship between the elastographic and viscosity data showed a positive moderate to high correlation (Figure 6).

Previous studies have indicated that the shear-wave velocity is sensitive to the probe position with respect to the direction of the fascicle plane. In a study of reproducibility of SWE values on the gastrocnemius medialis and tibialis anterior, Cortez et al. [28] demonstrated a fair-to-excellent interoperator reliability for measurements in the longitudinal plane, but a poor one for the transverse ones. The shear modulus in the longitudinal direction has been shown to be linearly related to passive and active muscle forces [29]. Romano et al. [14] described two protocols, one in a routine clinical setting and a dedicated protocol aiming for low muscle extension with statically significant lower variability, indicating the importance of precise member positioning in relaxed states. Similar to Romano et al., Lacourpaille et al. [30] described a protocol with various subject positioning using alternate degrees of muscle extension and reported good intra- and inter-observer reliability. Chino et al. [31] also obtained higher stability images in the longitudinal plane with a lower coefficient of variance (CV), showing better repeatability in the longitudinal measurements. This was the reason why we only took the measurements in a single location, in the longitudinal plane, avoiding vessels and fascia. In our study, for the deltoid muscle, patients were placed supine, with their elbow resting on a pillow and their arm bent at 90 degrees, a position that avoided passive stretching. However, for the soleus muscle, the prone position with knees fully extended presents a greater chance of passive stretching. We can hypothesize that as the overall stress–strain behavior of the muscle depends on fiber orientation and distribution, the viscoelasticity being an intrinsic parameter may generate anisotropy from a structural perspective, as both the elasticity and viscosity increase with contraction. The results from Looke et al. [11] suggest that the influence of the viscoelastic component is greater in the direction of the muscle fiber, as the fluid component moves easier along the fibers than across them. However, studying the relationship between viscosity and anisotropy is beyond the scope of this study; further studies are needed to give an answer to this inquiry.

An important limitation of our study is the rather small size of the sample (38 subjects). Ultrasound elastography, in general, is considered to be an operator-dependent technique. The development of various systems and the imaging methods available make homogenization difficult. However, the ICC showed excellent reproducibility between the measurements taken by the two sonographers, suggesting a very reproducible technique. There are other factors to consider, such as the reproducibility of the protocol; caution is needed when evaluating the same patients multiple times as reference values have to be clearly defined for a specific technical setup (the same machine, the same transducer, and, preferably, the same sonographer).

Dominance effects were not considered in our study, all the measurements were performed on the right arm and the right calf of the subjects.

Another limitation is using the curvilinear probe, as it was the only one available for the ViPLUS examination. High-frequency shear waves travel faster, so the measured viscosity of the muscle can also vary with frequency [1]. A possible technical limitation is reading the values in kilopascals, as they are susceptible to tissue heterogeneity and less precise compared to meters per second [32]. Further studies with linear transducers are mandatory to confirm these reference viscosity values and to assess the structural alterations in patients with musculoskeletal pathology.

## 5. Conclusions

The lack of standardization in muscle SWE measuring techniques still poses difficulty in using it as a reliable diagnostic method. Nowadays, a definitive diagnosis for neuromuscular diseases is based on muscle biopsy and genetic testing. However, for an experienced sonographer with a rigorously planned protocol, SWE and the novel ultrasound technique ViPLUS have great potential in monitoring the effectiveness of treatments, diagnosing muscle inflammatory disease. By quantifying the changes in the mechanical properties of the muscles, SWE and ViPLUS may become a reliable non-invasive biomarker.

## Figures and Tables

**Table 1:** Descriptive statistics of subjects’ demographics.

Variables	All Samples (n = 38) Median Range (Min–Max)	Male (n = 8) Median Range (Min–Max)	Female (n = 30) Median Range (Min–Max)
**Age (years)**	26 (21–29)	24 (21–28)	27 (21–29)
**BMI (kg/m^2^)**	20.98 (17.85–34.88)	25.78 (19.5–34.88)	20.5 (17.8–27.5)

Abbreviations: BMI, body mass index; n, total number of subjects; median range expressed as minimum–maximum values.

**Table 2:** Descriptive statistics of muscle mechanical properties.

Variables	Deltoid (Precontraction) Mean ± SD 95% CI of Mean	Deltoid (Postcontraction) Mean ± SD 95% CI of Mean	Soleus (Precontraction) Mean ± SD 95% CI of Mean	Soleus (Postcontraction) Mean ± SD Median (Range) 95% CI of Mean 25–75% Percentile
**SWE (kPa/s)**	22.2 ± 4.4 [21.2–23.2]	24.9 ± 5.3 [23.6–26.1]	13.1 ± 4.7 [12–14.1]	14.2 (4.2–31.7) [25% 11.3–75% 18.5]
**ViPLUS (Pa.s)**	2.9 ± 0.5 [2.8–3]	3.1 ± 0.5 [3–3.2]	2.1 ± 0.5 [1.9–2.2]	2.4 ± 0.6 [2.3–2.6]

Abbreviations: SWE, Shear-Wave Elastography; kPa/s, kiloPascal/second; ViPLUS, Viscosity Plane-Wave Ultrasound; Pa.s, Pascal.second; SD, Standard Deviation, CI, Confidence Interval.

**Table 3:** Paired group comparisons of elastographic and viscosity values between the pre- and post-contraction.

Variables	Deltoid SWE Comparison Pre/Post Contraction Paired *t*-Test	Deltoid ViPLUS Comparison Pre/Post Contraction PAIRED *t*-Test	Soleus SWE Comparison Pre/Post Contraction Wilcoxon Matched-Pairs Signed-Rank Test	Soleus ViPLUS Comparison Pre/Post Contraction Paired *t*-Test
*t*,df	4.72, 75	2.56, 75		4.48, 75
* *p* value (two-tailed)	**<0.0001**	**0.0124**	**0.0003**	**<0.0001**
r (correlation coefficient), *p* value (one tailed)	0.5, <0.0001	0.4, 0.0006	0.6, <0.0001	0.3, 0.0147

Abbreviations: SWE, Shear-Wave Elastography; ViPLUS, Viscosity Plane-Wave Ultrasound; *t*, *t*-value of the paired *t*-test; df, degrees of freedom. * *p* value is significant at <0.05 level.

**Table 4:** Multiple correlations between the elastography and viscosity values.

Correlation Coefficient [95% Confidence Interval] Significance Level *p* * (Two-Tailed)	Deltoid ViPLUS Precontraction	Deltoid ViPLUS Postcontraction	Soleus ViPLUS Precontraction	Soleus ViPLUS Post-Contraction
Deltoid SWE precontraction	Pearson r = 0.48 [95% CI 0.28–0.63] *p* < 0.0001			
Deltoid SWE postcontraction		Pearson r = 0.57 [95% CI 0.4–0.7] *p* < 0.0001		
Soleus SWE precontraction			Pearson r = 0.63 [95% 0.48 = 0.75], *p* < 0.0001	
Soleus SWE postcontraction				Spearman r = 0.75 [95% 0.63–0.83], *p* < 0.0001

Abbreviations: SWE, Shear-Wave Elastography; ViPLUS, Viscosity Plane-Wave Ultrasound; CI, Confidence Interval; r, Pearson/Spearman correlation coefficient; * *p* value is significant at <0.05 level.

**Table 5:** Results of the ICC calculations using a mean of two raters, an absolute agreement, and a two-way random-effects model [23].

Intraclass Correlation Coefficient							
	Intraclass Correlation ^b^	95% Confidence Interval		F Test with True Value 0			
		Lower Bound	Upper Bound	Value	df1	df2	Sig
Single Measures	0.912 ^a^	0.891	0.929	21.671	303	303	0.000
Average Measures	0.954	0.942	0.963	21.671	303	303	0.000

Abbreviations: df, degrees of freedom; Sig, significance. A two-way random-effects model where both the people and the measured effects are random. ^a^. The estimator is the same, whether the interaction effect is present or not. ^b^. Type A intraclass correlation coefficients using an absolute agreement definition.

## Data Availability

The data presented in this study are available on request from the corresponding author.
